# Sex differences in hypothalamic–pituitary–adrenal axis regulation after chronic unpredictable stress

**DOI:** 10.1002/brb3.1586

**Published:** 2020-03-10

**Authors:** Michelle C. Palumbo, Sky Dominguez, Hongxin Dong

**Affiliations:** ^1^ Department of Psychiatry and Behavioral Sciences Feinberg School of Medicine Northwestern University Chicago IL USA

**Keywords:** chronic stress, FKBP51, glucocorticoid receptor, HPA axis, sex differences

## Abstract

**Introduction:**

Exposure to stress, mediated through the hypothalamic–pituitary–adrenal (HPA) axis, elicits sex differences in endocrine, neurological, and behavioral responses. However, the sex‐specific factors that confer resilience or vulnerability to stress and stress‐associated psychiatric disorders remain largely unknown. The evident sex differences in stress‐related disease prevalence suggest the underlying differences in the neurobiological underpinnings of HPA axis regulation.

**Method:**

Here, we used a chronic unpredictable stress (CUS) model to investigate the behavioral and biochemical responses of the HPA axis in C57BL/6 mice. Animals were tested in the open field and forced swim test to examine anxiety‐like and depressive‐like behaviors. Plasma corticosterone levels were measured after behavior and CUS, and glucocorticoid receptor (GR) expression and cytosolic and nuclear fractions of binding protein FKBP51 expression were taken to measure function and regulation of the stress response.

**Results:**

Our results indicate increased depressive‐like behavior in males and females which correlated with increased corticosterone levels following CUS. However, females displayed more anxiety‐like behaviors with and without CUS. Interestingly, we found trends toward dysregulation of GR protein expression in CUS females, and an increase in the GR inhibitory protein, FKBP51, in the cytosol of CUS males but not females.

**Conclusion:**

These results suggest biochemical alterations to the HPA axis regulation which may elicit a glucocorticoid resistance in females after chronic stress and may contribute to the sex‐biased vulnerability to stress‐related psychiatric disorders.

## Graphical abstract

We found trends toward dysregulation of GR protein expression in CUS females, and an increase in the GR inhibitory protein, FKBP51, in the cytosol of CUS males but not females. These results suggest female susceptibility toward glucocorticoid resistance. This abnormal regulation of the HPA axis in females after chronic stress may contribute to the sex‐biased vulnerability to stress‐related psychiatric disorders.

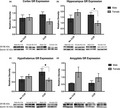

## 1 INTRODUCTION

Exposure to stress initiates a variety of behavioral, physiological, and cellular responses to prepare the body for alterations in homeostasis (Herman & Tasker, 2016; Stoney, Davis, & Matthews, 1987). The hypothalamic–pituitary–adrenal (HPA) axis mediates the autonomic, behavioral, and cognitive reactions of the stress response (Blank, Nijholt, Eckart, & Spiess, 2002; Orozco‐Cabal, Pollandt, Liu, Shinnick‐Gallagher, & Gallagher, 2006). Activation of the HPA axis in response to stress elicits the endocrine system to release glucocorticoids (GC), such as cortisol in humans, or corticosterone in rodents. Secretion of GCs from the adrenal cortex regulates the negative feedback mechanism through glucocorticoid receptors (GR) to reduce the activation of the HPA axis and terminate the stress response (Burke & Miczek, 2014). GRs are thought to mediate the feedback mechanism and therefore play a key role in maintaining HPA axis function (de Kloet, Joëls, & Holsboer, 2005).

In the unliganded state, the GR remains inactive in the cytoplasm in a multiprotein complex of heat shock and chaperone proteins (Pratt & Toft, 1997). Upon steroid binding, the GR translocates to the nucleus to regulate gene transcription and reduces corticotropin releasing factor (CRF) expression (Galliher‐Beckley & Cidlowski, 2009; Kageyama & Suda, 2009) and pro‐inflammatory cytokines (Rekers, de Fijter, Claas, & Eikmans, 2016). Recent work has indicated the immunophilin FK506‐binding protein 51 (FKBP51) is a glucocorticoid‐induced negative regulator of GR through sequestering the receptor into the cytoplasm and reducing hormone binding affinity (Davies, Ning, & Sánchez, 2005; Reynolds, Ruan, Smith, & Scammell, 1999; Stechschulte & Sanchez, 2011). Disrupting GR nuclear translocation has been postulated to lead to GC sensitivity, a characteristic found in preclinical and clinical populations of depression, where small traces of GCs can rapidly set off the HPA axis cascade (Denny, Valentine, Reynolds, Smith, & Scammell, 2000; Holownia, Mroz, Kolodziejczyk, Chyczewska, & Braszko, 2009; Westberry, Sadosky, Hubler, Gross, & Scammell, 2006; Woodruff et al., 2007). Additional evidence suggests impaired HPA axis function may be due to a disruption in number and function of GRs in the hippocampus and hypothalamus (Pariante, 2006). This “glucocorticoid resistance” is characterized by an over expression of CRF, hyperactivity of the HPA axis, and an inability of GRs to respond adequately to GCs. Animal and human studies suggest reduced expression and function of GRs may be relevant for the pathogenesis of stress‐related psychiatric disorders (de Kloet et al., 2005; Pariante & Lightman, 2008).

Men and women respond differently in physiological and neuroendocrine aspects of stress, which may influence the vulnerability or resilience of certain individuals to chronic stress (Ngun, Ghahramani, Sánchez, Bocklandt, & Vilain, 2011). Exposure to chronic stress also induces brain region‐specific and sex‐dependent neuronal activity alterations, which may play a role in the sexually dimorphic responses of the HPA axis (Franceschelli, Herchick, Thelen, Papadopoulou‐Daifoti, & Pitychoutis, 2014). One study found only female rats upregulate cochaperones that inhibit GR translocation and impair GC negative feedback (Bourke et al., 2013). Another found neurons of female rats are more sensitive to CRF and lack potential adaptive mechanisms found in male rats (Valentino, Bockstaele, & Bangasser, 2013), potentially mediating the neuroendocrine sex differences in HPA axis regulation.

Chronic exposure to inappropriate or sustained activation of the stress response is associated with the pathophysiology of numerous affective disorders (Bangasser & Valentino, 2014). Epidemiological data reveal sex differences in several affective disorders that are exacerbated by stress (Bangasser & Valentino, 2014). Many studies have examined how chronic stress contributes to the etiology of psychiatric disorders such as anxiety and depression (Bremne & Vermetten, 2001; McEwen, 2017; Pervanidou & Chrousos, 2018). The sex differences in disease prevalence suggests the underlying differences in stress‐related pathogenesis.

In this study, we investigated whether chronic unpredictable stress (CUS) would induce sex differences in affective behavior, corticosterone levels, GR protein, and FKBP51 expression levels. We explored potential mechanisms of sex‐biased GR activation and signaling after CUS in the modulatory regions of the HPA axis including the cortex, hippocampus, hypothalamus, and amygdala.

## 2 MATERIALS AND METHODS

### 2.1 Experimental animals

Male and female C57BL/6 mice (4–7 months) were assigned to either CUS or non‐CUS groups (*N* = 10–11). All mice were housed with 2–4 same‐sex littermates in plastic cages with bedding for the duration of the experiment unless otherwise noted and kept on a 12 hr light/dark cycle unless otherwise noted. Animals had access to food and water ad libitum*,* and the temperature was maintained at 22 ± 2°C. All care and use of animals were approved by Northwestern University's Institutional Animal Care and Use Committee in accordance with the NIH Guide for Care and Use of Laboratory Animals.

### 2.2 Chronic unpredictable stress (CUS)

Here, we adapted a model of CUS (Willner, 2005) to include a variety of “microstressors,” which vary in duration, intensity, and timing (Table 1). Our mild approach to CUS mimics chronic stress exposure as it relates to neuropsychiatric disorders and is extensively used to study animal models of induced anxiety and depression (Antoniuk, Bijata, Ponimaskin, & Wlodarczyk, 2019). This study randomly and variably performed multiple stressors to ensure unpredictability and lack of adaptation. We used a multimodal approach to CUS consisting of random, intermittent, and unpredictable exposure to a variety of stressors multiple times a day for 4 weeks (Table 2). Three randomly assigned stressors were given at variable times of the day for 2 weeks, followed by two stressors a day and an anxiety or depression task during weeks 3 and 4 (Table 3). Animals in non‐CUS treatment groups were left undisturbed in their housing units until behavioral testing began.

**Table 1:** The “microstressor” components of our chronic unpredictable stress (CUS) model in varying degrees of duration and intensity

Stressor	Duration
Cold water swim	3 min
Restraint	1 hr
Wet bedding	2 hr
No bedding	2 hr
Cage tilt	2 hr
Water deprivation	8 hr
Food deprivation	8 hr
Isolation	Overnight

**Table 2:** An example weekly schedule of the chronic unpredictable stress (CUS) paradigm used in this study

	Monday	Tuesday	Wednesday	Thursday	Friday	Saturday	Sunday
9 a.m.		Cold Water Swim		Restraint		Alter light/dark cycle	Alter light/dark cycle
10 a.m.	No Bedding				Water Deprivation		
11 a.m.			Cage Tilt				
12 p.m.							
1 p.m.		Wet Bedding			Cage Tilt		
2 p.m.				Cold Water Swim			
3 p.m.	Wet Bedding		No Bedding				
4 p.m.		Restraint					
5 p.m.				Food Deprivation	Restraint		
6 p.m.	Isolation		Isolation				

**Table 3:** The study's full timeline included four total weeks comprised of chronic unpredictable stress (CUS), two behavioral assays, and blood and tissue collection immediately following

Week 1	Week 2	Week 3	Week 4
CUS 3×/day	CUS 3×/day	CUS 2×/day	CUS 2×/day
		Open field test	Forced swim test
			Blood and tissue collection

### 2.3 Behavioral tests

Animals were habituated to the testing room for 1 hr prior to behavioral testing. All behavioral apparatuses were cleaned with 70% ethanol and deionized water to remove any previous animal scent.

#### 2.3.1 Open field

To examine anxiety and exploratory behaviors, animals were placed in the center of an open arena (72 × 72 × 36 cm) made of an evenly illuminated Plexiglas apparatus with a 3 × 3 lined center grid. A camera positioned above the arena recorded by video tracking system (Any Maze) for 10 min. Locomotor activity was automatically computed based on total distance traveled. Analysis was based on the time spent in the center of the arena or in the periphery.

#### 2.3.2 Forced swim

The forced swim test (FST) was carried out as a behavioral despair test and to assess depressive‐like responses. Animals were placed in a glass cylinder jar filled with water (±25°C) and allowed to swim freely for 6 min. Immobility is characterized by the absence of any horizontal or vertical movement excluding minor movements necessary for the animal to stay afloat during the last 4 min of the trial. The water was replaced after each usage.

### 2.4 Corticosterone assay

Retro‐orbital blood draws were performed immediately after the FST (*N* = 4–5 per group) to reflect the accumulation of CUS over the prolonged period. Once collected, plasma samples were immediately placed on ice, centrifuged at 25,200*g* for 20 min at 4°C, and the supernatant was collected and diluted for testing in the corticosterone ELISA following the manual's instructions (ENZO, ADI‐900‐097). The optical densities of reconstituted sample solutions were read at 405 nm in a plate reader (FUOstar Omega). Values are reported as adjusted values based on dilution factors and reported as pg/ml.

### 2.5 Tissue collection

After blood collection, the animals were put under anesthesia using pentobarbital and intracardially perfused with 0.1 M phosphate‐buffered saline (PBS). Brains were removed, dissected, and isolated into the hypothalamus, amygdala, hippocampus, and cortex for biochemical characterization. Brain subregions were immediately frozen at −80°C and stored until used for Western blot applications.

### 2.6 Sample preparation

About 20 mg of cortex tissue was used to separate lysates into nuclear and cytoplasmic fractions using a nuclear extraction kit (Epigentek, OP‐0002‐1) following kit protocol. Briefly, tissue was homogenized in 200 μL of NE1 and then allowed to incubate for 15 min followed by centrifugation for 10 min at 16,182*g* at 4°C. The supernatant was saved as the the cytoplasmic component, and the pellet was resuspended in 150–200 μL in NE2 for 15 min on ice with vortexing. This resuspension was then spun for 10 min at 21,952*g* at 4°C, and the supernatant was saved as the nuclear component. Both nuclear and cytosolic extractions were then measured for total protein concentration using a BCA protein kit assay (Pierce, TB263211).

### 2.7 Western Immunoblotting

We analyzed total protein concentrations of GRs (anti‐BuGR2) normalized against β‐actin and the cochaperone binding protein FKBP51 normalized against GAPDH (*N* = 4–6 per group). Due to tissue size and integrity, all extracted brain regions were used in GR expression quantification, while only frontal cortex homogenates were used to measure FKBP51 levels. Lysates were boiled for 10 min at 95°C and added to 5 µl loading dye (Millipore) for electrophoresis. The samples were separated on 10% SDS–PAGE gel (30 μg per sample) and then transferred to a polyvinylidene difluoride PVDF (Immobilan) membrane. PVDF membranes were blocked with 5% nonfat milk in TBS (20 mM Tris‐buffered saline, 0.2 M NaCl, pH 7.6) for 1 hr at room temperature and then incubated overnight on a shaker at 4°C with the primary antibodies against monoclonal BuGR2 1:1,000 (Thermo Fisher Scientific Cat# MA1‐510, RRID:AB_325427), monoclonal FKBP51 (D‐4) 1:200 (Santa Cruz Biotechnology Cat# sc‐271547, RRID:AB_10649040), monoclonal GAPDH 1:1,000 (Thermo Fisher Scientific Cat# AM4300, RRID:AB_2536381), and monoclonal β‐Actin 1:1,000 (Santa Cruz Biotechnology Cat# sc‐47778 HRP, RRID:AB_2714189) in TBS. After washing, membranes were incubated for 2 hr at room temperature with secondary antibodies anti‐mouse IgG Horseradishperoxidase (HRP) Conjugate 1:3,000 (Bio‐Rad) and then washed again. Immunmoblots were visualized chemiluminescently (ECL) with a detection system (PerkinElmer) using West Dura Extended Duration Substrate (Bio‐Rad) or SuperSignal West Femto (Thermo Fisher Scientific).

### 2.8 Image analysis

Imaging analysis software (ImageJ) was used to quantify all protein abundance as values of density intensity. All values were normalized against endogenous unaffected levels of β‐actin or GAPDH housekeeping proteins. If double bands were present around the anticipated site for a given protein, bands were averaged together and normalized against housekeeping proteins to account for individual loading differences or potential phosphorylation sites of each isomer.

### 2.9 Statistical analysis

Two‐way analyses of variance (ANOVA) were used to determine the effects of CUS on male and female C57BL/6 mice in behavioral and biochemical measures. All values are expressed as group means ± standard errors or the mean (*SEM*). Differences were considered significant at *p* < .05. All *post hoc* comparisons were conducted using Sidak's multiple comparisons tests. Data were analyzed using Prism 8.0 (GraphPad Software).

## 3 RESULTS

**Figure 1:**
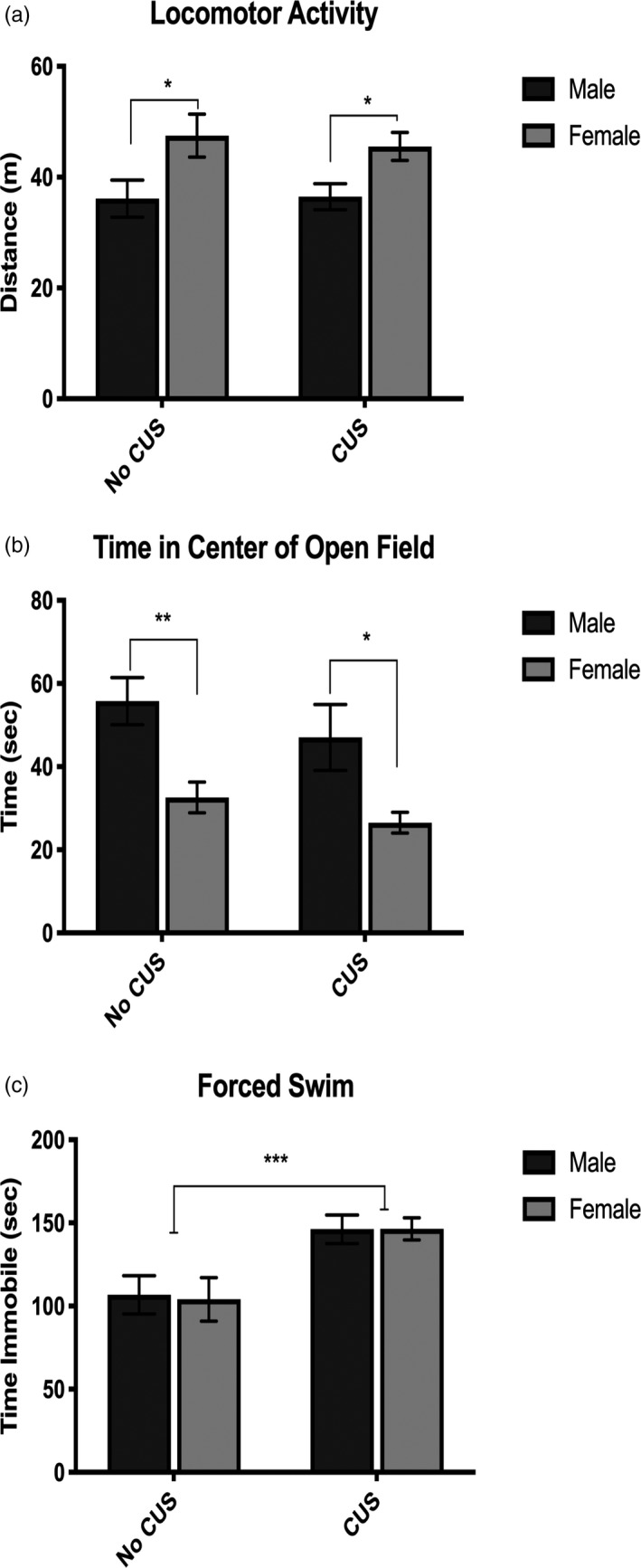
Behavioral analyses of anxiety‐ and depressive‐like behaviors in the open field and forced swim tests. Females display increased locomotor activity (a) and decreased time spent in the center of the open field arena (b). CUS induced depressive‐like behaviors in the forced swim test by increasing the time spent immobile in both sexes (c). *p < .05, **p < .01

Anxiety‐ and depressive‐like behaviors were assessed through open field and FST. CUS exposure did not affect locomotor activity or duration of time spent in the center of the arena in the open field test (Figure 1a,b). However, females in both groups displayed increased locomotor activity **(**1A) (*F*_1,38_ = 10.77, *p = *.0022) and spent less time in the center of the open field arena (1b) (*F*_1,36_ = 16.55, *p = *.0002), indicating sex differences in typical exploratory and anxious‐like behavior. In the FST, immobility behaviors are indicative of despair or depressive‐like behaviors, as mice are antagonistic toward water. CUS‐exposed animals spent significantly more time immobile compared with non‐CUS animals (1c) (*F*_1,37_ *= *16.07, *p = *.0003). Our results demonstrate CUS‐induced despair and depressive‐like behaviors in both sexes.

**Figure 2:**
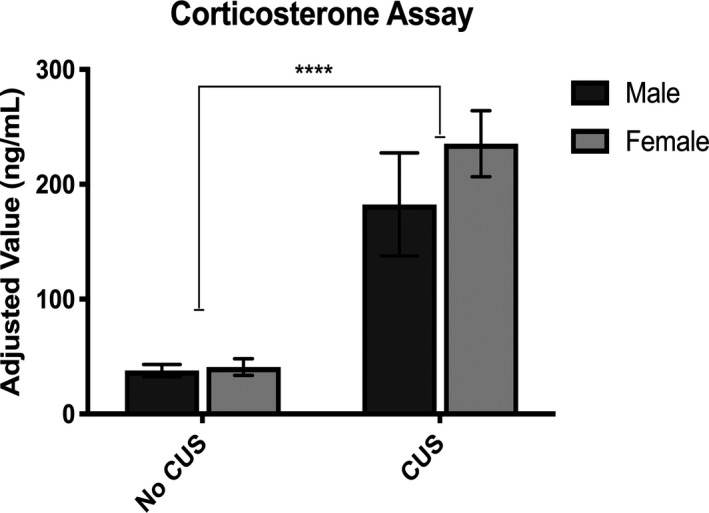
CUS induced elevated corticosterone plasma serum levels in both sexes compared with non‐CUS animals. ****p < .0001

Plasma was collected immediately after animals completed behavioral testing, and corticosterone levels were measured. Both CUS males and females displayed similar corticosterone concentration levels, which were significantly increased compared with non‐CUS mice (*F*_1,16_ = 112.6, *p* < .0001) (Figure 2). This suggests similar steroid production rates in both males and females.

**Figure 3:**
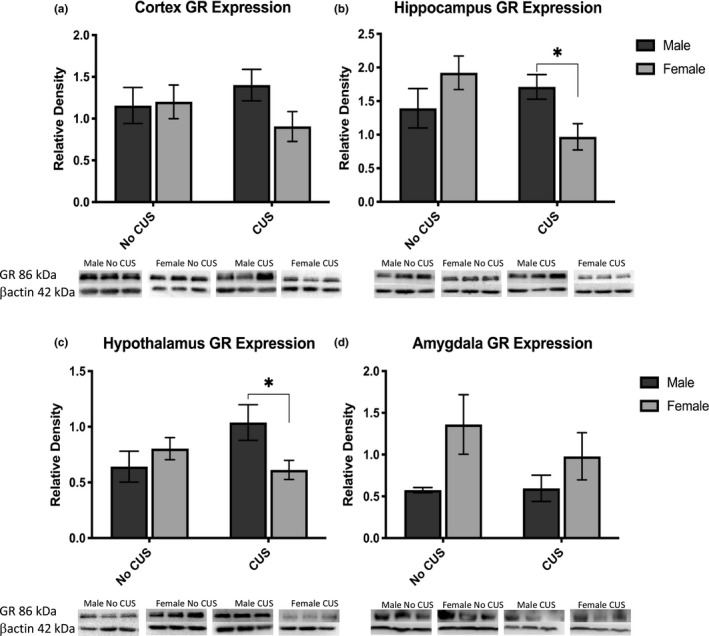
Glucocorticoid receptor expression normalized against bactin. Panels display functionally relevant brain regions of the HPA axis such as the cortex (a), hippocampus (b), hypothalamus (c), and amygdala (d) with respective immunoblots below each graph. *p < .05

To investigate sex differences in GR protein expression regulating the HPA axis negative feedback mechanism, we measured total protein GR levels using Western blot applications (Figure 3a–d). Two‐way ANOVA analysis determined a sex and stress interaction in GR levels in the hippocampus (*F*_1,16_ = 6.29, *p* = .0215) (3b) and the hypothalamus (*F*_1,20_ = 5.58, *p* = .0284) (3c). *Post hoc* comparisons confirmed CUS females decreased GR expression in the hypothalamus compared with CUS males (*F*_1,20_ = 2.420, *p* = .0497). GR expression in the hippocampus of CUS females was very close to reaching significance when compared to non‐CUS females (*p* = .0502). Similarly, GR expression in the cortex shows trends of significance between non‐CUS females and CUS females (*p* = .5513). The data in the cortex, hippocampus, and hypothalamus demonstrate a clear pattern of decreased GR expression in CUS females, while GR expression in CUS males remained relatively unchanged. In comparison, both CUS and non‐CUS females had an upregulated GR expression in the amygdala (*F*_1,18_ = 4.707, *p* = .0437) (3d), although *post hoc* analysis did not show significant difference between groups, possibly due to relatively small numbers in each group (*N* = 4–6). Nevertheless, our results suggest CUS females have a compromised negative feedback regulation in functionally relevant brain regions of the HPA axis.

**Figure 4:**
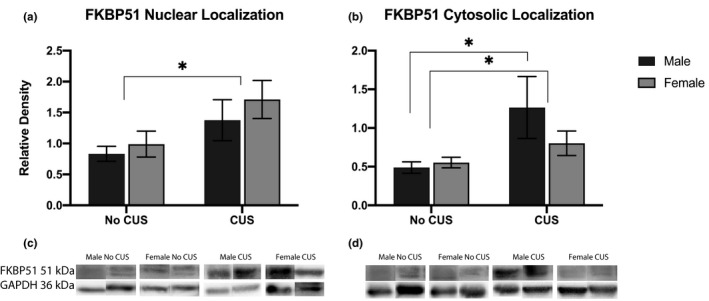
Cochaperone protein FKBP51 expression normalized against GAPDH. Panels display cortex homogenates separated into nuclear localizations (a) and cytosolic localizations (b) with their respective immunoblots, (c) and (d), below each graph. *p < .05

To further explore sex‐specific regulation of GR signaling in the stress response, we measured FKBP51 expression in nuclear and cytosolic fractions of the frontal cortex (Figure 4). We found an effect of stress with CUS males and females increasing nuclear FKBP51 expression (*F*_1, 35_ = 6.288, *p = *.0169) (4a). Additionally, we found an effect of stress in cytosolic FKBP51 expression (*F*_1, 35_ = 6.083, *p = *.0187), driven by CUS males increasing cytosolic fractions from non‐CUS males (*F*_1, 35_ = 2.603, *p = *.0268), while CUS females had no significant change from non‐CUS females (4b). Given the inhibitory properties of FKBP51, these findings may potentiate an inhibition of GR down‐stream processing in males, while an aggregation of GR in the cytosol for females, potentially leading to glucocorticoid resistance.

## 4 DISCUSSION

Differences in biological sex may induce differential coping, adaptive, and signaling mechanisms in response to aversive events. These alterations may promote sex‐specific vulnerabilities to stress‐related disorders, characteristic of an over active HPA axis and inability to mediate the stress response feedback loop. However, the mechanisms underlying the sex‐specific responses to stress remain largely unknown.

In the present study, non‐CUS animals appeared to have basal corticosterone levels which indicates a proper HPA axis attenuation immediately after behavioral testing experience, when the blood was drawn. Blood collection times were aimed to reflect the accumulation of CUS over the 4‐week experiment. The given CUS aversive environment elevated plasma corticosterone levels in both sexes indicating sustained HPA axis activation. Sex differences in corticosterone may exist with exposure to acute stress, but chronic stress may physiologically affect both sexes in a similar fashion (Anderson et al., 2019). Some studies of CUS report elevated plasma corticosterone only in females (Dalla et al., 2005), while others report no significant differences between sexes (Duncko, Kiss, Skultétyová, Rusnák, & Jezová, 2001; Grippo et al., 2005). Inconsistencies in stress paradigms, duration, timing of plasma collection, and sample preparation for a given assay of choice can impact the resulting quantitative measures. However, with proper control groups, the relative differences when compared against treatment groups is most telling data. One limitation of this study does not control for circulating hormone levels by using ovariectomized females. Hormonal alterations may contribute to neuronal alterations in the stress response (Weathington & Cooke, 2012). However, using male and female littermates, in CUS and non‐CUS groups, represented animals of various estrus cycle stages as best as experimentally possible. Overall, we show long‐term exposure to CUS resulted in sustained elevated plasma corticosterone levels in both sexes compared with non‐CUS animals who were left alone in their homecages until behavioral testing. Moreover, it is evident that corticosterone dysregulation has sex‐specific implications in the functional regulation of the HPA axis.

This study used an adapted model of CUS known to induce a constellation of sex‐specific neurochemical alterations including dopaminergic, serotonergic, and noradrenergic changes (Xing et al., 2013) that may play a critical role in the neural processing of the stress response. Surprisingly, CUS did not affect locomotor behavior in either sex possibly due to the habituation of novel situations in the CUS paradigm. However, we show females in both groups increased locomotor activity and decreased time exploring the center of the open field arena compared with males. Perhaps, females already have a higher basal anxious‐like behavior compared with male mice and their behavior cannot be amplified more by exposure to CUS. Thus, sex differences in exploration and movement will remain consistent for CUS and non‐CUS groups. Our results are in concordance with other stress paradigms in the open field test where total movement did not differ between sexes (Duncko et al., 2001) and female mice initially demonstrated the most anxious behavior by spending the least amount of time exploring in the center of the open field arena (Dalla et al., 2005; Dong & Csernansky, 2009).Exposure to CUS increased despair and depressive‐like behaviors in both sexes in the FST. Due to the great physiological stress of the FST, it was the last given behavioral test and thus reasonable why both sexes in the CUS group should display despair‐like behaviors. Although it is possible to be a confounding factor, the experimenters postulated the stark water temperature differences between the 4°C cold water swim in the CUS paradigm and the 25°C FST should not be an issue. The given behavioral assays may be valid to evaluate general tendencies of behavior but may not be specific enough to differentiate between underlying mechanisms of the stress response, and thus, biochemical analyses should give a better insight into the neurobiological effects of CUS.

Clinically depressed populations report females who exhibit higher cortisol levels take a longer time to return to baseline levels and may show a decrease in number and function of GRs to modulate and respond appropriately to the over expression of CRF (Weinstock, Razin, Schorer‐Apelbaum, Men, & McCarty, 1998). Several brain regions and endocrine glands work in concert to modulate stress through the HPA axis. The corticolimbic circuitry of the stress response comprised of the amygdala, prefrontal cortex, and hippocampus mediates the emotional reactivity from stressful events, such as fear and anxiety (Adhikari et al., 2015). The hippocampus has some of the highest levels of GR in the brain and serves as a slow negative feedback mechanism (Conrad, 2008). GRs in the cortex are also functionally important in cognitive‐related processing in the fear response circuit (Adhikari et al., 2015). Together, the central responses of stress to GRs in the cortex have been known to modulate the HPA axis and the hippocampus (Gold, 2015). Given the regulation and modulation of GRs in the cortex, hippocampus, hypothalamus, and amygdala in the HPA axis, this study chose to investigate these regions for GR protein expression levels.

Here we show, CUS downregulated female GR expression in three functionally critical regions of the HPA axis. Similar reduction patterns in the cortex, hippocampus, and hypothalamus of CUS females indicate an inadequate HPA axis regulation in the presence of CRF over expression. Without proper receptor quantities in place, CUS females are limited in modulating a sustained stress response, suggesting CUS females may be experiencing a “glucocorticoid resistance.” This resistance results in a biological inability to respond to elevated circulating GC levels, thus may increase the production of GCs. Although females have higher amygdala GR expression compared with males; surprisingly, there were no significant changes in female GR levels after CUS in the amygdala. The increase in female amygdala GR expression may be a compensatory mechanism to attenuate the global stress tolerance. Reduced GR expression has been shown to limit the effects of GC by mitigating the negative feedback mechanism, resulting in more GCs to be released into the blood stream (Miller & O'Callaghan, 2005). Since the CRF gene and protein expression is negatively regulated by GCs, it is possible that GR reduction may play a role in the upregulation of CRF after CUS (Herman & Tasker, 2016). Thus, a reduction in GR expression in multiple brain regions from CUS females could be due to an inability, or resistance, of GRs to adequately respond to a jeopardized HPA axis regulation. Clinical studies investigating this theory suggest the main neuroendocrine abnormality in depressed patients is a lack of functional response and abated levels of GR (Boyle et al., 2005; Pariante, 2006). Taken together, the reduction of GR expression in female mice may be a potential compensatory mechanism aimed to overcome the GC resistance.

In order to better understand the sex‐specific mechanistic signaling of GRs in CUS‐induced mice, we investigated the expression and localization of the cochaperone binding protein, FKBP51 in the frontal cortex. In the absence of a ligand, the cytosolic localization of GR remains inactive but exhibits a high ligand affinity. The GR sits in a multiprotein complex comprised of chaperones and immunophilins, including members of the FK506 family such as FKBP51 and FKBP52 (Cain & Cidlowski, 2015). Upon GC binding, the GR undergoes conformational changes and post‐translational modifications allowing it to translocate to the nucleus for gene transcription (Jenkins, Pullen, & Darimont, 2001). Here, we report CUS males and females increased nuclear FKBP51 expression, with CUS females displaying the highest nuclear FKBP51 levels. In the cytosol, our results show both CUS genders increased FKBP51 expression; however, these numbers are largely driven by CUS males, which were significantly greater compared with non‐CUS males. This interesting result lead us to question the biological mechanism which may be driving this phenomenon. Several reports have characterized FKBP51 as a strong inhibitor of GR function (Fries, Gassen, Schmidt, & Rein, 2015; Riggs et al., 2003), reducing transcriptional activity, (Wochnik et al., 2005) and delaying nuclear translocation of the receptor. Given the inhibitory properties of FKBP51, our results suggest male mice react to CUS with an adaptive response to GC over expression in the cytosol and nucleus, while females have an inadequate reaction by only increasing FKBP51 in the nucleus. The lack of proper inhibitory mechanisms in the cytosol for females under CUS as the ligand binds to the receptor may be one of the first maladaptive mechanisms putting females at risk for a dysfunctional GC attenuation. On the other hand, the male compensatory mechanism of increased FKBP51 in both the nucleus and the cytosol may assist in regulating CUS through decreasing GC sensitivity. These sex differences in GR binding protein localization suggest neurobiological differences in the mechanistic response and function of GR binding.

Some reports of chronic stress in mice indicate increased FKBP5 expression (Lee et al., 2010), others report decreased levels of *FKBP5* mRNA and FKBP51 protein (Volk et al., 2016). However, these studies omit female mice completely, removing any possibility of sex differences in FKBP51 function or expression, a critical factor in GR regulation and action. Despite the sex differences in affective and stress‐related disorders, the female sex remains commonly excluded from clinical and preclinical studies (Zucker & Beery, 2010). This study gives a novel insight to potential sex differences in FKBP51 expression, which may be a leading factor for GR resistance. It is hypothesized that depressed patients have increased basal levels of FKBP51 (Lukic et al., 2015; Tatro et al., 2009) and has become an important target for physiological stress regulation and potential new drug target therapies for patients with major depressive disorder (Binder et al., 2004; Kirchheiner et al., 2008; Stamm et al., 2016).

In summary, we found exposure to CUS affects HPA axis regulation in a sex‐dependent manner. Importantly, females may lack the ability to tolerate and mitigate the stress response due to downregulated GR expression and deficiency of FKBP51 binding protein expression in the cytosol. Persistent and potentially compromised HPA axis activation in females could lead to high incidences of stress and psychiatric disorders known to worsen due to chronic stress. Ultimately, the sustained elevated corticosterone levels could have secondary physiological effects such as an increase in inflammation, oxidative stress, or neurodegeneration. Studying sex‐specific mechanisms in the stress response can contribute to the improvement of diagnosis and effective individualized treatment of stress‐related disorders such as PTSD, major depression, and anxiety disorders.

## CONFLICT OF INTERESTS

None of the authors have any conflicts of interest to declare.

## AUTHOR CONTRIBUTION

MP maintained animal colonies; collected plasma and tissue; ran behavioral assays, corticosterone assays, and immunoblots; and performed data analysis and writing of the manuscript. SD ran FKBP51 immunoblots and analysis. HD contributed to intellectual troubleshooting, funding, and all equipment and space necessary for this project. All authors contributed to manuscript editing.

## Data Availability

The data that support the findings of this study are available from the corresponding author upon reasonable request.
